# Human papillomavirus vaccine uptake among adolescent girls living with HIV in Côte d’Ivoire: A national cross-sectional survey

**DOI:** 10.1016/j.vaccine.2026.128434

**Published:** 2026-03-11

**Authors:** Simon Boni, Maëva Quessette, Elise Nedelec, Evelyne Akotangni, Danielle Ackah, Dao Siaka, Julie Jesson, François Eboua, Georges Tiahou, Valeriane Leroy, Madeleine Amorissani-Folquet, Antoine Jaquet

**Affiliations:** a University of Bordeaux, National Institute for Health and Medical Research (INSERM) UMR 1219, Research Institute for Sustainable Development (IRD) EMR 271, Bordeaux Population Health Centre, Bordeaux, France; b Programme PACCI, Treichville, Abidjan, Côte d’Ivoire; c Programme National de Lutte contre le Cancer, Abidjan, Côte d’Ivoire; d Université Paris Cité and Université Sorbonne Paris Nord, IRD, Inserm, Ceped, 75006 Paris, France; e ONG Centre Solidarité Action Sociale (SAS) de Bouaké, Côte d’Ivoire; f Université de Toulouse, Inserm, CERPOP, Toulouse, France; g Service de Pédiatrie, Centre Hospitalier Universitaire (CHU) de Yopougon, Abidjan, Côte d’Ivoire; h Direction de la Formation et de la Recherche en Santé, Ministère de la Santé, de l’Hygiène Publique et de la Couverture Maladie Universelle, Abidjan, Côte d’Ivoire; i Service Pédiatrie, Centre Hospitalier Universitaire (CHU) de Cocody, Abidjan, Côte d’Ivoire

**Keywords:** Cervical cancer, HPV vaccination, HIV, Adolescents, West Africa

## Abstract

**Background:**

Human papillomavirus (HPV) vaccination programs are scaling-up in low-and-middle-income countries, targeting girls aged 9 to 14, usually through a school-based approach. However, specific data on adolescent girls living with HIV (AGLHIV) are lacking. We estimated the HPV vaccine uptake and its determinants among AGLHIV in Côte d’Ivoire.

**Methods:**

A nationally representative survey was conducted within HIV pediatric facilities from July 2024 to February 2025 through a mixed-method approach. Participating facilities were selected through a hybrid sampling combining the inclusion of the largest pediatric HIV facilities and a random sample of integrated HIV facilities. Vaccine uptake was documented from immunization records, and questionnaires to AGLHIV. A logistic regression analysis was used to assess factors associated with HPV vaccine uptake. In-depth interviews were conducted with AGLHIV, parents/guardians, and healthcare workers (HCW) to assess barriers to HPV vaccine uptake.

**Results:**

Overall, 659 AGLHIV (median age 15 years [IQR: 13–17]) were included across 15 HIV facilities. Among participants, 97 (14.7%) received at least one dose of HPV vaccine. Reported barriers to vaccination included: lack of information about the vaccine (85.4%), parents’ refusal (8.9%) and fear of adverse events (5.7%). Identifying HPV as a risk factor for cervical cancer (adjusted Odds Ratio (aOR) = 4.02 [95% CI: 1.87–8.62] having ever attended a focus group on sexual education (aOR = 1.79 [95% CI: 1.07–2.99] and receiving care outside of Abidjan (aOR = 3.07 [95% CI: 1.88–5.01]) were independently associated with vaccine uptake. In-depth interviews highlighted a lack of information, stigma, parental influence, and a lack of vaccine proposition by HCW as barriers to HPV vaccination.

**Conclusion:**

The HPV vaccine uptake was low among AGLHIV in Côte d’Ivoire. Person-centered interventions combining school- and facility-based approaches, with dedicate monitoring tools, are urgently needed to achieve the WHO 90% HPV vaccination coverage among AGLHIV.

## 1. Introduction

Human papillomaviruses (HPV) are the most common infectious cause of malignancy in adult women around the world, and account for around 73% of infectious related cancers in West Africa [1]. Cervical cancer (CC) is the most common and the deadliest HPV-related cancer among women in developing countries [2]. Although this infection is mainly sexually transmitted and affects approximately 75% of sexually active women early in their sexual life, only 5–10% of infections persist and lead to cancers in case of persistent high-risk HPV (hr-HPV) infection in the absence of screening [3]. Over 200 HPV types are known to infect human genital organs, of which 12 types are classified as oncogenic to humans (HPV16/18/31/33/35/39/45/51/52/56/58/59) [4]. In Côte d’Ivoire, the overall prevalence of hr-HPV in women living with HIV of childbearing age was estimated to 54%, higher than the 33.7% prevalence reported in their HIV-uninfected counterparts [5]. Limited information is available in Adolescents, Girls living with HIV (AGLHIV) aside a previous study reporting a 3% prevalence of hr-HPV in a sample of 250 AGLHIV with a median age of 13 years, highlighting the relevance of implementing HPV vaccination in this vulnerable group [6]. As a global response, the World Health Organization (WHO) approved the use of HPV vaccines for girls 9 to 15 years old, before initiating their sexual activity [7]. From traditional vaccines like Gardasil-4, Gardasil-9, and Cervarix-2; new vaccines have been developed recent years with the potential of reduced costs and higher opportunity for low-and middle-income countries to launch HPV vaccines within their expanded immunization program [8,9]. In 2025, 155 (80%) of the 194 WHO member States have introduced HPV prophylactic vaccines in their national programs [10,11]. In 2024, WHO shifted the two doses recommended to a single-dose for adolescent girls aged 9 to 14 years based on growing evidences supporting the non-inferiority of this reduced vaccine schedule [12]. However, immunosuppressed girls are recommended to receive at least two doses and complete the full three-dose vaccination schedule when possible, in the absence of sufficient data supporting schedules reduction [8]. The WHO global strategy to accelerate the elimination initiative has committed governments to rollout interventions to reach 90% of young girls before 15 [7]. AGLHIV are exposed to multiple health risks, have specific sexual and reproductive health needs, and facing challenges when transitioning to adult care [13,14]. Compared to their HIV-uninfected peers, they are facing higher risk of persistent hr-HPV infection, and HPV-related diseases including CC [15,16].

Since 2019, Côte d’Ivoire has implemented a routine HPV vaccination program, mainly school-based delivered to girls aged 9 (and 10–14 through catch-up campaigns), free-of-charge at a national level. Vaccine was administered equally to all eligible girls, regardless of their HIV status [17]. Additional catch-up campaigns were done twice at national level, and sporadically by health counties since the scale up of the program. In August 2023, the country has endorsed the WHO and the national immunization technical advisory group recommendation on HPV vaccine single-dose, except in AGLHIV [18]. The 2024 National CC prevention guidelines did not recommend HPV vaccination for boys, mainly due to financial resource constraints and aimed at ensuring the sustainability of HPV vaccination for girls [19].

The WHO recommends to monitor HPV vaccine coverage by dose and by age [20]. In 2025, the HPV vaccination uptake, estimated through the health districts activities’ report, was 62–65% for the first dose and 47% for full doses in global population, with disparities across the continent and considered populations [10,21]. In Côte d’Ivoire, the national HPV vaccine single dose coverage in all eligible-girls was 61% in 2023, with no stratification according to HIV status [10]. However, data on AGLHIV are scarce. Less is known about the adherence to HPV vaccination for AGLHIV for whom the three-dose schedule is still required.

We estimated the HPV vaccine uptake and explore the individual- and system-level factors driving decision-making for vaccination among AGLHIV in Côte d’Ivoire.

## 2. Methods

### 2.1. Study design & setting

A cross-sectional mixed method study was conducted within pediatric HIV care facilities at a national level in Côte d’Ivoire from July 2024 to February 2025.

In Côte d’Ivoire, pediatric HIV care is integrated into a three-tier health system comprising primary, secondary, and tertiary levels. Aside from these integrated public facilities, are HIV clinics with largest cohorts and more structured services, coordinated by NGOs. These pediatric HIV care facilities are distributed across all regions of the country.

A total of 15 national HIV pediatric care facilities were selected through a hybrid sampling approach. Major health facilities delivering HIV care services to AGLHIV in Abidjan, the economic capital, as well as in four other regions of the country, participated in this study (Supplemental file 1). First, a convenience approach was used by including the largest HIV pediatric facilities in the country. These facilities included those contributing to the IeDEA West Africa pediatric network in Côte d’Ivoire, delivering care in Abidjan [22]. They were “The CePReF – Centre de Prise en Charge, de Recherche et de Formation of Yopougon”; “The CIRBA – Centre International de Recherche Bioclinique d’Abidjan”; “The CMSDS – Centre Medical de Suivi des Donneurs de Sang”; and the HIV pediatric units from University Hospitals of Cocody and Yopougon. They demonstrated robust competencies in HIV care, data collection and monitoring and outcomes of treatment for their HIV populations (children, adolescents, or adults) [22]. An additional HIV program named “The Centre SAS” was added. This HIV clinic, led by a Non-Governmental Organization is specialized in the comprehensive care of people living with HIV and their families, with a particular focus on pediatric, adolescents and youth care. The organization operates in 9 regions and 25 health districts, covering approximately 25% of the country’s health regions. Its operational branch in Bouake and Korhogo were particularly involved in enrolling participants in Center and Northern regions [23]. Finally, based on the national HIV registry obtained from the National HIV/AIDS program, we performed a systematic random sample based on the list of integrated facilities providing HIV pediatric care and having reported at least 15 young girls and adolescents over a consecutive three-month in 2023, in each region of the country.

### 2.2. Population

All AGLHIV aged 9 to 19 years at January, 1st 2024, infected with HIV (HIV-1, HIV-2, or HIV-1&2), in care in one of the participating sites were solicited to participate in the study. They were requested to participate the day of their antiretroviral therapy (ART) refiling or after a phone call inviting them in the clinic for a dedicated study visit. Exclusion criteria included AGLHIV with instable hemodynamic conditions, unable to answer questions, and those who had moved to another region compromising their attendance at the participating health facility. At site level, the local research team composed of community health workers (CHW), the main healthcare provider (physician or midwife/nurse) and the research assistant collaborated to inform and invite eligible AGLHIV.

### 2.3. Data collection

A standardized questionnaire under REDCAP software [24], was administered to AGLHIV aged 9 to 19 and/or their parents/legal guardian, separately through a 20-min face-to-face interview in a private room within the HIV clinic. Data were collected on sociodemographic characteristics (age, education, employment status, access to internet), sexual and reproductive health, knowledge about CC and HPV, HPV vaccine awareness, and history of a HPV vaccine proposition and uptake, as well as barriers to HPV vaccine uptake. In settings where AGLHIV were unable to speak French, questions were translated in local language to ensure the accuracy of responses. A final interview was done with the parent/guardian to confirm the HPV vaccine uptake and provide the reference (with the date) and the number of doses received by the AGLHIV. Prior to the study, healthcare workers (HCW) at the clinic were trained to study procedures and eligibility criteria. They assessed and confirmed study eligibility, and asked AGLHIV and or their parents/legal guardian to participate in the study.

*In depth interviews were* led by two social science researchers and one trained assistant, carried out between April and November 2024. The first phase of interviews took place in Abidjan, followed by fieldwork in three other regions outside the economic capital. In-person and audio recorded in-depth interviews were conducted with AGLHIV, parents or legal guardians, and healthcare professionals directly involved in HIV pediatricians and adolescent ‘care; national-level cancer and immunization leaders as well as policy makers. Interviews among AGLHIV and their parents continued until thematic saturation defined as the point at which no emerging information or theme were obtained through additional interviews, was reached.

### 2.4. Outcome definition

The main outcome was “HPV vaccine uptake”, defined as having ever received at least one HPV vaccine dose prior to the study. The declaration was confirmed by a proof of vaccination which was mainly the immunization card delivered by the national immunization program or an ascertainment of the parent/guardian statement after describing circumstances (period, place, procedures) of the vaccination. In case where the HPV vaccine card was not available, the circumstances of vaccination were described to determine whether or not the HPV vaccine had been received. The HPV vaccine uptake was disaggregated according to the number of received doses (none, 1, 2 or 3).

### 2.5. Statistical analysis

The quantitative component purposely generated descriptive and analytical data. Categorical variables, including HPV vaccine uptake, were described using frequencies and their proportions, while continuous were described as median, and their interquartile range (IQR). Comparisons were made using Chi2 square or Fischer exact tests when applicable, for categorical variables.

A logistic regression model was used to assess factors associated with HPV vaccine uptake. Adjustment variables were mode of HIV transmission, time since first HIV diagnosis, educational level of AGLHIV and her mother/legal guardian, HIV clinic location (Abidjan or outside), CD4 count and HIV viral load at enrollment in care and at the time of the survey, a proposition of HPV vaccine at school or in community outreach or in any health facility, prior awareness of CC and HPV infection. Crude and adjusted Odds ratio with their 95% confidence Intervals (95% CI) were calculated during bivariable and multivariable analyses, respectively. A stepwise descending procedure was applied; and the age group (9; 10–14 and 15–19 years old) was forced in the final model. All quantitative analyses were done using *Stata Statistics/Data Analysis* version 14.1.

Regarding the qualitative component, data collection and analysis were carried out iteratively, with constant comparison of data to ensure a theoretical saturation. Data were examined using content analysis based on grounded theory [25].

We employed a combination of conceptual frameworks: The Health Belief Model (HBM) and the 5C model to examine the barriers to and facilitators of HPV vaccination. The HBM emphasizes the influence of perceptions of susceptibility, severity, benefits, and barriers on vaccine decisions [26,27]. The 5C model complements this by identifying confidence, complacency, constraints, calculation and collective responsibility as the main psychological factors influencing vaccination behavior [28]. Combining these two frameworks allowed a thorough analysis that links individual beliefs with social and contextual determinants of HPV vaccine uptake.

### 2.6. Ethical aspects

An approval was obtained from the National ethical committee for science and life *(Ref 239–23/MSHPCMU/CNESVS-km)* in Côte d’Ivoire. Prior to their enrolment, participants were informed about the study purpose, procedures, and complete the informed consent process. AGLHIV aged 18–19 years provided a sign consent form while the youngest were requested to provide a signed assent form. Parents/guardians provided a signed consent form.

## 3. Results

### 3.1. Study population

Over the study period, 667 AGLHIV were invited though their parents to participate in the study, of whom 8 refusals were notified. Overall, 659 AGLHIV with a median age of 15 years [IQR: 12–17)], located in Abidjan for 50.1% (*n* = 330), were included. Among them, 23 (3.5%) had no formal education, 204 (31%) were at the primary level and the remaining 432 (65.5%) reached the secondary school level. No access to internet was reported for 286 (43.4%) participants Table 1.

HIV was perinatally-acquired for 568 (86.2%) AGLHIV. AGLHIV were in care since a median time of 10 [IQR 6–12] years, with WHO clinical stage I/II for 73.7% at enrolment in care (*n* = 486).

*A total of 50 in-depth interviews were conducted including* 22 AGLHIV, 13 parents or legal guardians, 11 healthcare professionals directly involved in HIV pediatricians and adolescent ‘care; and 4 policy makers and health programs implementers or coordinators Table 2.

The enrolled AGLHIV were characterized by a diverse socio-demographic profile. In the semi-urban/rural areas, most AGLHIV parents or caregivers had no formal education or primary school level. Healthcare professionals participating in the study exhibited a wide range of profiles, encompassing doctors, nurses, midwives, etc.

### 3.2. HPV vaccine uptake

Among participants, 97 (14.7%) had received at least one dose of HPV vaccine: 82 (12.4%) one dose; 12 (1.8%) two doses and three (0.4%) three doses. HPV vaccine uptake was higher in AGLHIV from facilities outside Abidjan (19.4%) compared to facilities located in the urban area of Abidjan (7.5%), *p* < 0.001 Fig. 1.

The venues for vaccination were school for 73 (75.3%), HIV care facility for 12 (12.4%), other HIV facility different from their HIV care center for 2 (2.1%) and community for 10 (10.3%). AGLHIV from facilities outside Abidjan received HPV vaccine mainly in school (85.1%) followed by HIV clinic (6.0%), while those from Abidjan received it in school (53.3%) and then in HIV clinic (26.7%); *p* = 0.007.

In multivariate analysis, AGLHIV followed up outside Abidjan (aOR = 3.28 [95% CI: 1.69–5.74], having ever attended a focus group on sexual education (aOR =1.79 [95%CI: 1.07–2.99], and identifying HPV infection as a sexually transmitted infection (aOR = 3.64 [95% CI: 1.66–7.99] were more likely to get vaccinated compared to others. Parent or legal guardian awareness about CC was also independently associated to HPV vaccination uptake, after adjusting on factors mentioned above (aOR = 2.3 [95%CI: 1.28–4.28] Table 3.

Most of AGLHIV interviewed *(n* = *12/22; 54.5%)* reported not being afraid of the pain associated with injection, as they regularly undergo blood tests as part of their routine medical follow-up. They were also more likely to accept vaccination, as the fear of experiencing a double burden of disease is strongly present.

“Well, I can say, with the illness I have, that I’m a bit fragile. So, if there’s the possibility, I’ll do it without a problem.” — AGLHIV, Abidjan

In addition, the involvement of religious leaders in improving the vaccine’s acceptability is considered as a significant facilitator by health professionals.

### 3.3. Barriers to HPV vaccine uptake

Main reported barriers to HPV vaccination by AGLHIV were a lack of information about vaccine (85.4%) and fear of adverse effects (5.7%); lack of parental approval or consent (8.9%) and doubt about the vaccine effectiveness (0.9%) Fig. 2.

After counseling, 500 out of the non-vaccinated AGLHIV (89%) were willing to be vaccinated once the opportunity will be present.

The lack of awareness was the most common barrier to HPV vaccine uptake, reported by AGLHIV from all settings of the study, and even among schooled ones.

“During the interviews, I realized that they were unaware of the existence of this vaccine. Because normally, teenage girls receive the vaccine in CE2 and CM1 (3^rd^ and 4^th^ Grades, elementary school) classes, which are the classes that are usually scheduled for this. But most of them said that they had never attended an HPV vaccination session.” – Nurse, HIV pediatric care unit, Abengourou

The lack of information was closely linked to associated fears and therefore source of anxiety, emergence of rumors, notably the belief that the vaccine’s purpose is to make women ill or sterile. This rumor was found both in Abidjan, and outside particularly in semi-urban or rural settings (from one AGLHIV per setting).

“Some people are reluctant. This is because they haven’t been given the information. They believe that the CC vaccine can make girls infertile. So I tell them outright that this isn’t true. These are myths designed to discourage people from getting vaccinated, but it’s important to get vaccinated.” – Community health worker, Bouaké

“We are Africans; we really love having children. So, when you want to vaccinate a 9-year-old girl, the parents think it makes you sterile. Yes, that’s the first information they get.” — Nurse, PNLCa, Abidjan

The final decision regarding vaccination uptake was mostly made by parents. However, AGLHIV often attended their HIV follow-up appointments alone.

“So, when I go to school, and then, well, the educators talk about the vaccines, that some people are going to come. My parents say not to do it. Because we don’t know what it is.” — AGLHIV, Abidjan

One AGLHIV disagreeing with her parent regarding the decision for HPV vaccination. She would have taken proactive steps to protect her health by getting vaccinated if she had had the opportunity.

“When I come to the hospital like this, I like it. Because I want what is good for me…Yes, but all of them did it, and I didn’t…My parents, no. They didn’t want me to” - AGLHIV, Abidjan

The lack of time during medical attendances with AGLHIV to inform them about the HPV vaccine was reported by two healthcare professionals. This time constraint often resulted in focusing on ART renew and counseling for adherence to; investigation of potential complications), rather than preventive aspects such as HPV vaccination.

“That [HPV vaccination] isn’t our priority. Our priority is HIV. […] It’s not easy to discuss it [HPV vaccination] individually during a consultation, because we have a lot of patients and other things to do!” – Pediatrician, HIV clinic, Abidjan

Stigma was retrieved as concern for completing vaccine schedules in AGLHIV. The fear of disclosing HIV status and facing stigmatization from other adolescents and even family members was common.

“Because of my illness, they say I have to take lots of vaccines. But if I take too many, they [adolescents and family members] will suspect that I have HIV.” - AGLHIV, Daloa

Moreover, 9-year-old girls represent a new target group for routine vaccination, which modifies the behavioral patterns surrounding the vaccination process. From healthcare professionals particularly in rural areas perspective, guidelines and objectives for HPV vaccination are still unclear, leading to misunderstandings about the target population, as well as confusion regarding the leadership in coordinating HPV vaccination coordination (HIV or routine vaccination or cancer national programs).

The impact of the COVID-19 pandemic was also felt in the simultaneous rollout of the HPV vaccine. The pandemic heightened fears and generated confusion among the population.

“Regarding vaccination. Yes, after COVID I had some hesitations, huh. Because those who got vaccinated against COVID, there were effects, we heard about side effects. So because of that, others say: ‘ah, aren’t they going to have the same thing? Is it effective? Is it, is it… lots of questions.’” —Pediatrician, HIV Clinic, Abidjan

Severe shortages of the HPV vaccine have also been reported by two healthcare workers and three others stakeholders from national health programs and implementing partners, which reinforced existing barriers and limited vaccination outreach activities.

“That’s what tires the parents a bit, or sometimes when they go, they’re told there’s no antigen. They have to come back, and they don’t come back…” —Pediatrician, HIV Clinic, Abidjan

“Unfortunately, we are lacking vaccines. Today, in terms of demand, the population is willing to accept them. But on the supply side, we don’t have vaccines. There is always a lingering tension.” Stakeholder, International partner, Abidjan

### 3.4. Awareness towards HPV and CC

Among AGLHIV, 295 (44.8%) and 73 (11.1%) reported ever heard about CC and HPV infection, respectively. Overall, 170 (25.6%) participants were aware about HPV vaccine (26.4% in Abidjan versus 25.2% outside Abidjan), and 164 (24.9%) have received a proposition for HPV vaccination: 26.1% in Abidjan and 23.7% outside (*p* = 0.485). The information supporting the proposition of HPV vaccination was from health professionals (21.2%), school teachers and administration (21.3%), medias including social medias (15.9%), CHW in HIV clinics (6.1%), and others (12%).

Almost all of AGLHIV and their parents/guardians were familiar with the word “cancer”‘ but they did not necessarily associate it with a clear definition.

Specifically, about CC, AGLHIV and their parents had limited knowledge. Only one AGLHIV and two parents knew what it was, how it occurs and knew little information about it. Outside of Abidjan, only a few schooled AGLHIV had heard about the HPV vaccine. Their main sources of information were TV, internet, social networks, focus group and advice received at the hospital.

Two out of three specialist HCW had limited knowledge about the vaccine targets, delivery approach and schedules. Greater awareness of HPV vaccination has been observed in other HCW and its importance.

“There is a school strategy involving the national education authority. Teachers are also involved. Initially, the school health service and the service dedicated to adolescents and young people are involved” – Stakeholder, PNLCa, Abidjan

“I don’t know much about it yet” –Pediatrician, HIV clinic, Abidjan

“I would say it’s a vaccine used to prevent CC. It is intended for girls aged 9 to 14, and two doses are required.” –Nurse, HIV pediatric care unit, Abengourou

A need to improve vaccine awareness and training among healthcare providers was also reported by six responders.

“I think it’s already a matter of raising awareness and informing providers about the benefits of the vaccine. If we don’t have the information, … we won’t be able to pass on the information to the beneficiaries.” – Doctor, HIV clinic, Bouaké

## 4. Discussion

This nationally representative study showed a low HPV vaccine uptake among AGLHIV in 2025. Being followed in a HIV care facility outside Abidjan, a prior participation to a focus group on sexual education, identifying HPV as a STI, were identified as facilitators. Main reported barriers included a limited information about HPV vaccine, doubt about its effectiveness and the fear of adverse effects, parental influence, stigma, severe shortages in HPV vaccine and the lack of HPV vaccine proposition by health professionals in HIV pediatric care.

Our study demonstrated that less than one-fifth of AGLHIV received at least one dose of HPV vaccine in Côte d’Ivoire, although a previous study demonstrated the appropriateness of the HPV vaccination program regarding the distribution of hr-HPV types contributing to invasive CC, irrespective of HIV status [29]. This estimate is far from the reported 61% coverage of HPV vaccination in general population, which is based on aggregated data through the Dhis2 system [10]. The caveat between these estimations poses the need of reevaluating through accurate survey with robust methodology the coverage of HPV vaccination in both HIV-uninfected adolescent girls and AGLHIV. Despite the decentralization and affordability of the HPV immunization program, AGLHIV remain a vulnerable population, facing challenges accessing preventive intervention outside HIV. Our findings highlighted barriers comparable to those observed among vaccine-eligible populations, regardless of HIV status, as previously documented in in Malawi [30]. In the absence of differentiated delivery strategies, AGLHIV share structural and logistical challenges hindering the implementation of HPV vaccination. Indeed, vaccine shortages in the country have led to delayed national mass campaigns. Frequent shortages encountered by the national immunization program, also did not allowed regular campaigns organization. The urgent pressure on vaccine manufacturing industries during the COVID-19 pandemic, combined with the increasing number of countries implementing HPV vaccination at the same time probably resulted in challenges access vaccine supplies. This could have slow down Côte d’Ivoire momentum in preparing nationwide and more inclusive HPV vaccination campaigns. Côte d’Ivoire experiences in HPV vaccination for girls irrespective of their HIV status, showed a decrease in coverage since 2019 [10], also demonstrated by a WHO-UNICEF vaccination coverage estimates [31]. AGLHIV who are more vulnerable might have been more exposed to persistent challenges in rumors spread during the pandemic. Indeed, despite efforts to inform and promote, distrust of the HPV vaccine has persisted [30]. The COVID-19 pandemic has been identified as barriers to HPV vaccination in Sub-Saharan Africa [32]. A study from Kenya reported a decrease in HPV vaccine confidence and a low uptake for second dose (16%) in young girls during the COVID-19 pandemic [33]. Parents and girls might have mixed HPV vaccine and COVID-19 vaccine in a context of rumors and doubts surrounding the rapid emergence of the COVID-19 vaccine. While the country succeed to maintain the routine immunization in that period, school-based and community outreach campaigns with a potential to reach AGLHIV were postponed [34]. Shortages in HPV vaccines, already reported in Malawi [30] impacted routine awareness and vaccine delivery activities at school, potentially explaining that some AGLHIV declared not being aware about HPV vaccine. Specifically, AGLHIV face limited access to HPV vaccination due to the constraints of school-based delivery strategy. Outside the school setting, vaccination opportunities declined, while limited healthcare provider information and vaccine unavailability in HIV clinics that further contributed to missed opportunities for HPV immunization. Unlike a previous report [35], stigma related to sexuality or HIV was not identified as a barrier to accessing the first dose, but the subsequent ones.

A previous single-facility based study in AGLHIV from Uganda reported a low vaccine uptake and suggested Individual and structural barriers [36]. HIV-related care surrounding AGLHIV and their caregivers, adolescent leadership and cultural contexts were also reported in South Africa and in Malawi as key drivers to HPV vaccine uptake [30,37]. The public communication on HPV (infection and vaccine) seemed insufficient to efficiently reach AGLHIV parents and guardians. In addition, as highlighted by key informants, the limited information available at HIV clinics or integrated care facilities resulted in missed opportunities for HPV vaccination. Parents could lack information on HPV vaccine, hence a limited parent approval when schooled AGLHIV asked for consent prior the HPV vaccination. The limited information on HPV vaccine and CC was reported in other African contexts, as the main barrier to vaccination [38–41]. Our findings have emphasized the importance of improving public awareness to target parents, with the adjunction of a specific community-based intervention embedded in HIV facilities to reach AGLHIV. This implies reinforcing the knowledge of HCW to enable them to provide accurate information to AGLHIV, while also identifying AGLHIV peers to promote HPV vaccination. Literacy on HPV infection, risk of CC and vaccine as a preventive tool was associated with HPV vaccine uptake in AGLHIV, as previously reported in Central Uganda in addition to a previous sexual experience [42]. These elements support the need and benefits to improve literacy on HPV infection in AGLHIV to enhance vaccine uptake.

The close linkage to the healthcare system has been revealed as a key facilitator of vaccine uptake in AGLHIV. Our study suggests that health education through safe and trusted platforms, such as focus groups on sexual education, is a strong enabler of HPV vaccination. The misunderstanding about the vaccine effectiveness and tolerability, as well as the parental hesitancy could be less important. The confidence in vaccine should therefore be raised after a massive targeted information, given by HIV care providers and community counsellors, themselves appropriately trained on HPV vaccines and CC. The health system should take advantages of the regular medical appointments of AGLHIV by ensuring a continued vaccination offer through the HIV clinic and better connect these clinics to county-level coordination of HPV and cancer interventions. AGLHIV followed outside Abidjan might be closer to community health workers, hence a facilitated connection to school and community-based interventions. The community and stakeholders’ engagement have been demonstrated as facilitators to HPV vaccine [32,36]. In addition, AGLHIV outside Abidjan might be less likely to be engaged in social medias discussions where rumors and doubts on vaccine are common. Their main referent in vaccine information is their HCW whom they trust, hence higher rate of vaccination. The most common venue of information about HPV vaccine in HIV-uninfected girls (school-based) seems less efficient to enhance the uptake of HPV vaccine in AGLHIV. Therefore, engaging CHW is essential to improve HPV vaccination coverage. AGLHIV and their parents need information and reassurance about the tolerability of HPV vaccines [42]. Acceptability of HPV vaccine, once a clear information is provided was high in our study. The willingness to be immunized against HPV was noted in nine out of ten AGLHIV with no previous history of vaccine, once informed. In Western countries, data on uptake in AGLHIV is scarce, but the overall HPV vaccination uptake varies considerably across settings [10,43]. High vaccine cost, cultural barriers, the COVID-19 pandemic, the regular CC screening participation by mothers and safety concerns contributed to decrease adhesion to HPV vaccine [44].

Waiting for the adoption of single-dose HPV vaccination in immunocompromised girls given the promising ongoing studies such as the HOPE II study [45], the second and third doses’ rates were worryingly low, compared to a report from Uganda with 22% and 8% of uptake, respectively [36]. This suggests that HPV vaccination services, as currently provided is less appropriate for AGLHIV. Nakibuuka et al reported the inadequacy of HPV vaccination services at the hospital and in the schools as a barrier to vaccination for AGLHIV in Uganda [36]. Indeed, well-organized catch-up strategies for HPV vaccination program are needed. A differentiated approach in HPV vaccine delivery is urgently needed to close the gap on completed HPV vaccine schedule. Strategies that involve AGLHIV themselves as peer educators for newly diagnosed AGHIV who are eligible for HPV vaccine could be encouraged, as already suggested by AGLHIV in Malawi [30]. A synergistic strategy with the routine school-based approach could be leveraged by strengthening the first dose uptake in schools using tailored sensitization, and by replacing schools’ leaders and parents in HPV vaccine promotion especially in high urbanized cities such as Abidjan. As shown in Eswatini, Malawi and Uganda [46], stigma is a key barrier to HPV vaccination schedule in AGLHIV as receiving second and third doses over one (for HIV-uninfected girls) could raise suspicions with higher risk of HIV status disclosure. For this reason, AGLHIV should be encouraged to receive their first dose in existing general population platforms for HPV vaccination, and complete their scheduled vaccination in their HIV care facilities. Ultimately, if well implemented, this approach could help reach the WHO target of 90% fully vaccinated girls before 15 years-old by 2030 [47], while highly contributing to vaccine equity in sub-Saharan Africa.

Our study has some limitations. The HPV vaccine uptake relied on participants’ declarations for one-third of AGLHIV, with potential social desirability and information bias, leading to possible over-estimation of HPV awareness and vaccine uptake. This declaration was considered accurate when AGLHIV (or their parents) reported having received an injectable vaccine in their school during a campaign or routinely, or in communities. The HPV vaccine is the only vaccine offered to AGLHIV in such circumstances, before and after the COVID-19 pandemic. Additionally, the cross-sectional design of the survey did not allow the evaluation of the casual relationship between individual or system related factors and HPV vaccine uptake. Despite these limitations, our study is among the first documenting challenges in HPV vaccine uptake in West Africa, in a context of global HPV vaccination roll-out. The multi-county and the mixed of urban and semi-urban settings reinforce the generalizability of the findings to all AGLHIV in the country. The adjunction of the social science perspectives reinforces the value of our findings. We were able to better analyze data by triangulating both quantitative and qualitative data, particularly when addressing barriers to HPV vaccine uptake. Findings could inform countries highly affected by both HIV and HPV to leverage national efforts towards CC elimination in high-risk population such as AGLHIV.

Three months after the end of our study (April 2025), Côte d’Ivoire launched a national school and community-based HPV vaccination campaign with a catch-up program of adolescents up to 18 years old. We hope that the present survey has raised awareness towards HPV in participating HIV clinics and incited more AGLHIV to get vaccinated. In the view of WHO CC elimination by 2030, subsequent surveys using such methodology should be promoted to monitor the progresses, evaluating new strategies and efficiently address the persistent barriers.

## 5. Conclusion

Despite the decentralization and affordability of the immunization program, uptake of the HPV vaccine was worryingly low among AGLHIV in Côte d’Ivoire in 2024. A comprehensive CC awareness program is needed to address barriers to vaccination and target AGLHIV, as well as their parents and caregivers. Initiatives involving HIV facilities are also required to achieve the WHO target of 90% HPV vaccination coverage among AGLHIV. Person-centered interventions combining school- and facility-based approaches are urgently needed to improve HPV vaccine uptake in this vulnerable population; together with the development of dedicate monitoring tools to better track progress towards vaccination coverage.

## Supplementary Material

Supplementary files 1_2

## Figures and Tables

**Fig. 1.**
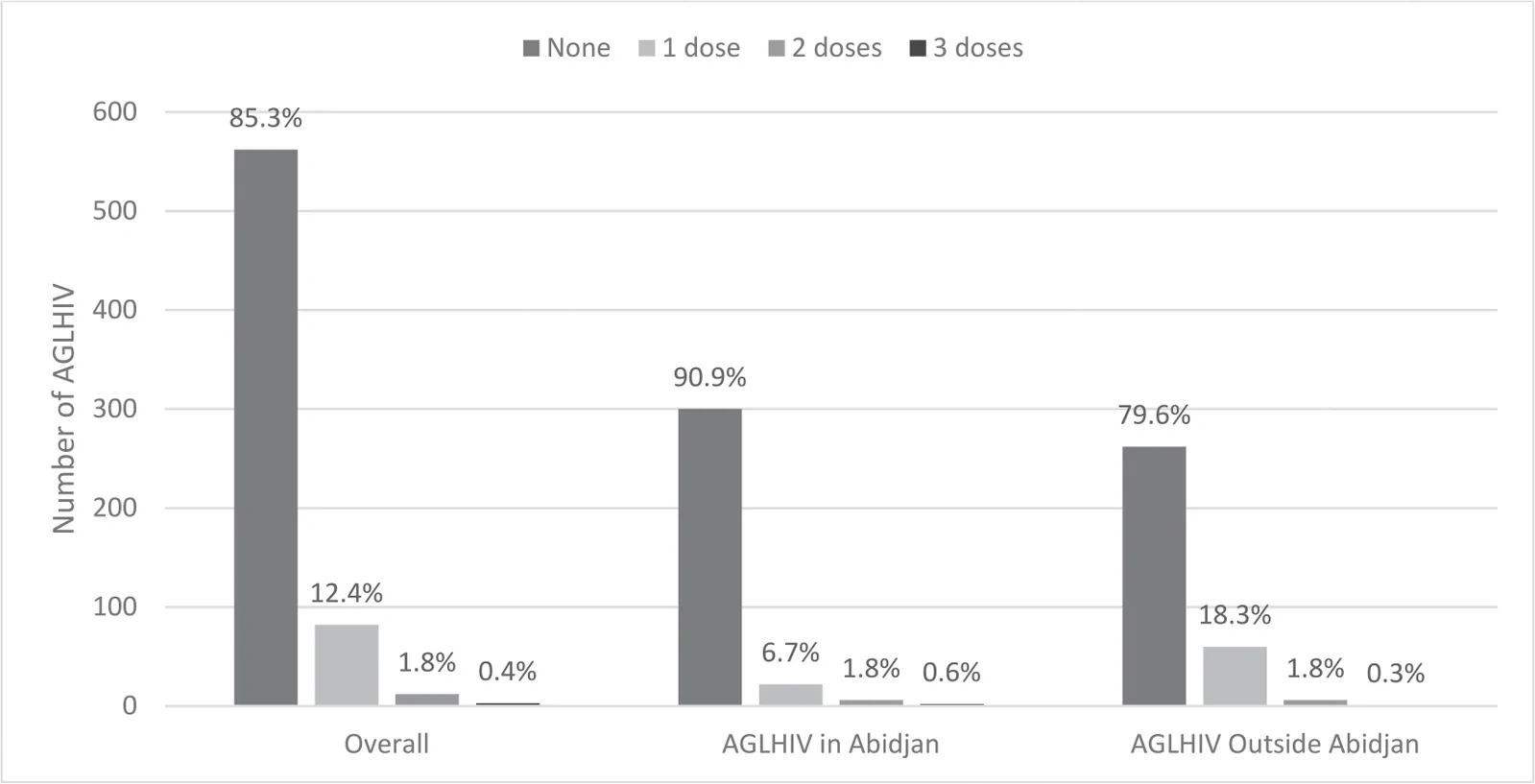
HPV vaccine received according to the location of the HIV care facility in 2025 (*n* = 97). *Percentages were calculated in each strata of AGLHIV regarding the location of the health facility delivering HIV care.

**Fig. 2.**
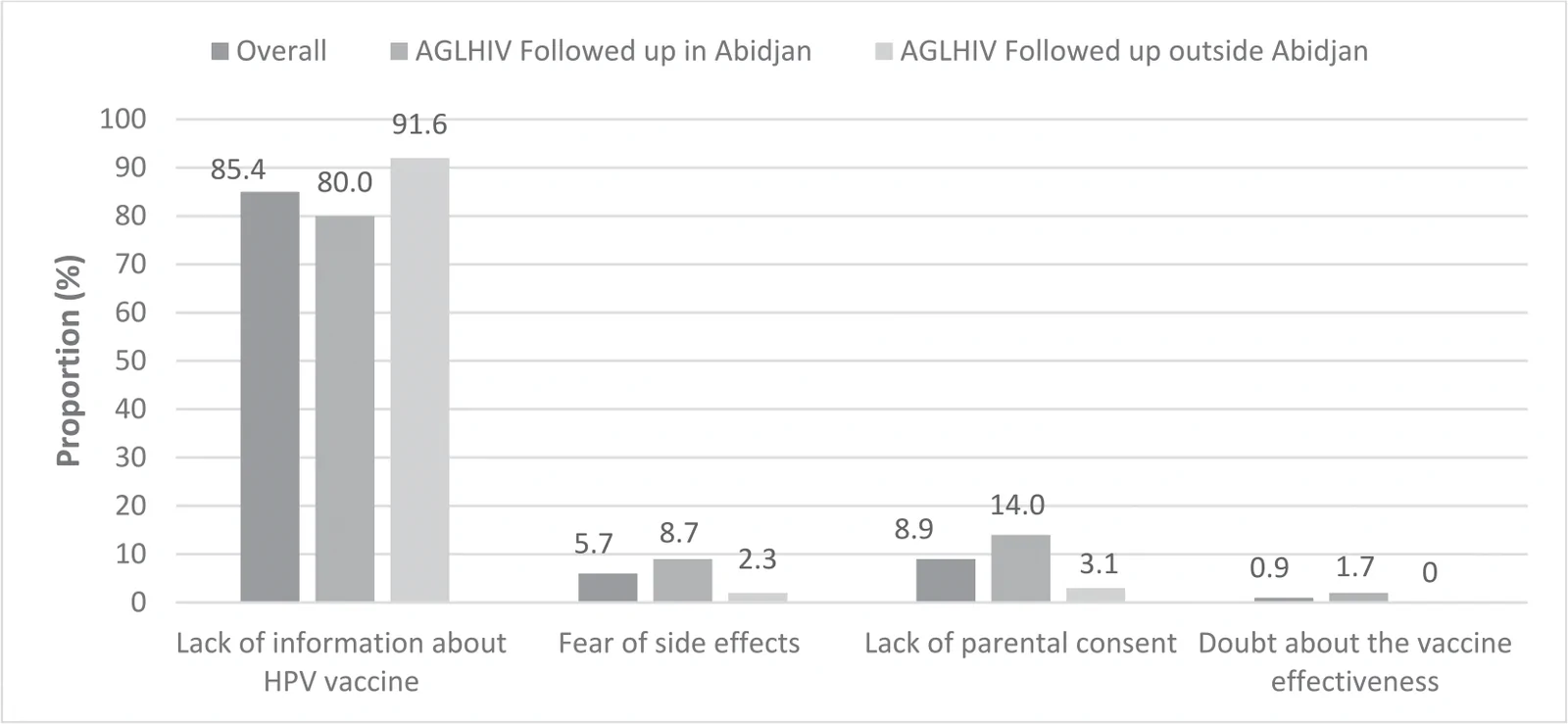
Reported barriers to HPV vaccine uptake in AGLHIV with no HPV vaccine uptake in Côte d’Ivoire, 2025 (*n* = 562).

**Table 1:** General characteristics of AGLHIV in Côte d’Ivoire, *COVADOCI study (N = 659)*.

Characteristics	Total N = 659		Having received at least one HPV vaccine dose *N* = 97; 14.7%		Not vaccinated N = 562; 85.3%		*P* value
				
	n	(%)	n	(%)	n	(%)	

**Age,** median [IQR] year	15 [13–17]		15 [12–17]		15 [13–18]		0.042
9	23	(3.5)	0	(0.0)	23	(4.1)	
10–14	266	(40.4)	46	(47.4)	220	(39.1)	
15–19	370	(56.1)	51	(52.6)	319	(56.8)	
**Educational level**							0.284
No formal/Primary	227	(34.5)	34	(35.0)	193	(34.3)	
Secondary –1st cycle^¥^	305	(46.3)	51	(52.6)	254	(45.2)	
Secondary-2nd cycle	109	(16.5)	10	(10.3)	99	(17.6)	
University	18	(2.7)	2	(2.1)	16	(2.9)	
**Access to internet**							0.642
Never	286	(43.4)	38	(39.2)	248	(44.1)	
< once per month	45	(6.8)	7	(7.2)	38	(6.8)	
>once per month but <1 per week	37	(5.6)	4	(4.1)	33	(5.9)	
At least once per week	111	(16.9)	21	(21.7)	90	(16.0)	
>5 per week	180	(27.3)	27	(27.8)	153	(27.2)	
**Use of contraceptives during the last 12 months** ^*^							0.886
No	588	(89.3)	88	(90.7)	500	(88.9)	
Yes	49	(7.4)	7	(7.2)	42	(7.5)	
Not willing to answer	22	(3.3)	2	(2.1)	20	(3.6)	
**Type of contraceptive method (*N* = 49)**							0.104
Mechanic	12	(24.5)	0	(0.0)	12	(28.6)	
Hormonal	37	(75.5)	7	(100.0)	30	(71.4)	
**Tobacco use**							0.283
Never	653	(99.1)	95	(97.9)	558	(99.3)	
Current	1	(0.2)	0	(0.0)	1	(0.2)	
Former	5	(0.8)	2	(2.1)	3	(0.5)	

* Among all the participants.

¥ In Côte d’Ivoire, the secondary school level start at 6th level to the year of baccalaureate. It is divided to two levels: fist cycle (the four first years) and second cycle (the three last years).

**Table 2:** Interviews breakdown by region and quality of key informants.

Category of informants	Abidjan	Outside Abidjan	Topics covered in the interviews

AGLHIV	15	7	Knowledge and perceptions of cancer in general, cervical cancer, HPV and vaccination; information on sexual and reproductive health. Facilitators and barriers to vaccine uptake
Parents/legal guardian	8	5	Knowledge and perceptions of cancer in general, cervical cancer, HPV and vaccination. Facilitators and barriers to vaccine uptake
Healthcare professionals	5	6	Knowledge and perceptions about HPV vaccination; experience of HPV vaccination; facilitators, challenges, feasibility
Policy makers	4	–	Knowledge and perceptions about HPV vaccination; strategies for key population (AGLHIV); facilitators, challenges, feasibility
**Total**	**32**	**18**	

**Table 3:** Factors associated to HPV vaccination uptake in AGLHIV in Côte d’Ivoire in 2025, *COVADOCY study (N = 659)*.

Characteristics	HPV vaccination uptake (Yes)	Bivariable analysis			Multivariable analysis		
	
		OR^*^	95% CI	p	aOR^*^	95% CI	p

**Age of AGLHIV**	**n (%)** ^*^			0.665			
9–10	9 (14.5)	1			1		0.29
11–14	37 (16.0)	1.17	(0.85–2.01)		0.91	(0.39–2.08)	
15–19	51 (14.0)	0.96	(0.44–2.06)		0.68	(0.29–1.61)	
**Education level of AGLHIV**				0.265			
No/primary level	34 (15.0)	1					
Secondary-1st cycle	51 (16.7)	1.14	(0.71–1.82)				
Secondary-2nd cycle or University	12 (9.5)	0.59	(0.29–1.19)				
**Internet access**				0.277			
Never	38 (13.2)	1					
<1 per week	11 (13.4)	1.01	(0.49–2.07)				
≥ 1 per week	48 (16.5)	1.28	(0.81–2.04)				
**Parent/legal guardian educational level**				0.825			
No/Primary	59 (15.7)	1					
Secondary/University	30 (13.4)	0.83	(0.52–1.33)				
Missing	8 (8.2)	0.86	(0.38–1.91)				
**Having attended ever a focus group on sexual education**				0.219			
No	44 (13.1)	1			1		0.026
Yes	53 (16.5)	1.31	(0.85–2.02)		1.79	(1.07–2.99)	
**Having sexual discussion with family/close relatives**				0.967			
No	62 (14.8)	1					
Yes	35 (14.6)	0.99	(0.63–1.55)				
**Parent/legal guardian aware of CC**							
No	15 (8.3)	1			1		0.041
Yes	78 (17.4)	2.31	(1.29–4.14)	0.005	2.34	(1.28–4.28)	
Not applicable^**^	4 (4.1)	1.69	(0.52–5.50)	0.381	1.40	(0.42–4.66)	
**AGLHIV identifying HPV as CC aetiology**				<0.001			<0.001
No/not wish to answer	84 (13.5)	1			1		
Yes	13 (35.1)	3.47	(1.70–7.08)		4.02	(1.87–8.62)	
**HIV care facility location**				<0.001			<0.001
Abidjan	30 (9.1)	1			1		
Outside Abidjan	67 (20.4)	2.56	(1.61–4.05)		3.07	(1.88–5.01)	

CC: Cervical cancer AGLHIV: Adolescents Girls living with HIV; CI: Confident Interval; OR: Odds Ratio; aOR: Adjusted OR.

* Percentage of AGLHIV with HPV vaccination uptake per strate.

** AGLHIV attending alone the HIV care facility.

## Data Availability

The data for this study are available, and can be provided from PAC-CI (www.pac-ci.org) upon reasonable request.

## Appendix A. Supplementary data

Supplementary data to this article can be found online at https://doi.org/10.1016/j.vaccine.2026.128434.

## Abbreviations

AGLHIV: Adolescent’ Girls Living with HIV
ART: Antiretroviral treatment
CC: Cervical cancer
CHW: Community Healthcare Workers
COVID-19: Coronavirus disease 2019
HIV: Human Immunodeficiency Virus
HPV: Human PapillomaVirus
OR: Odds Ratio
aOR: Adjusted Odds Ratio
WHO: World Health Organization
