# Supplementary material for: Human papillomavirus vaccine uptake among adolescent girls living with HIV in Côte d’Ivoire: A national cross-sectional survey
Source: Vaccine. Author manuscript; Available in PMC 2026 Nov 4. (PMC13733359; doi:10.1016/j.vaccine.2026.128434)
Supplement: Supplementary file 1 [file NIHMS2217313-supplement-Supplementary_files_1_2.pdf]

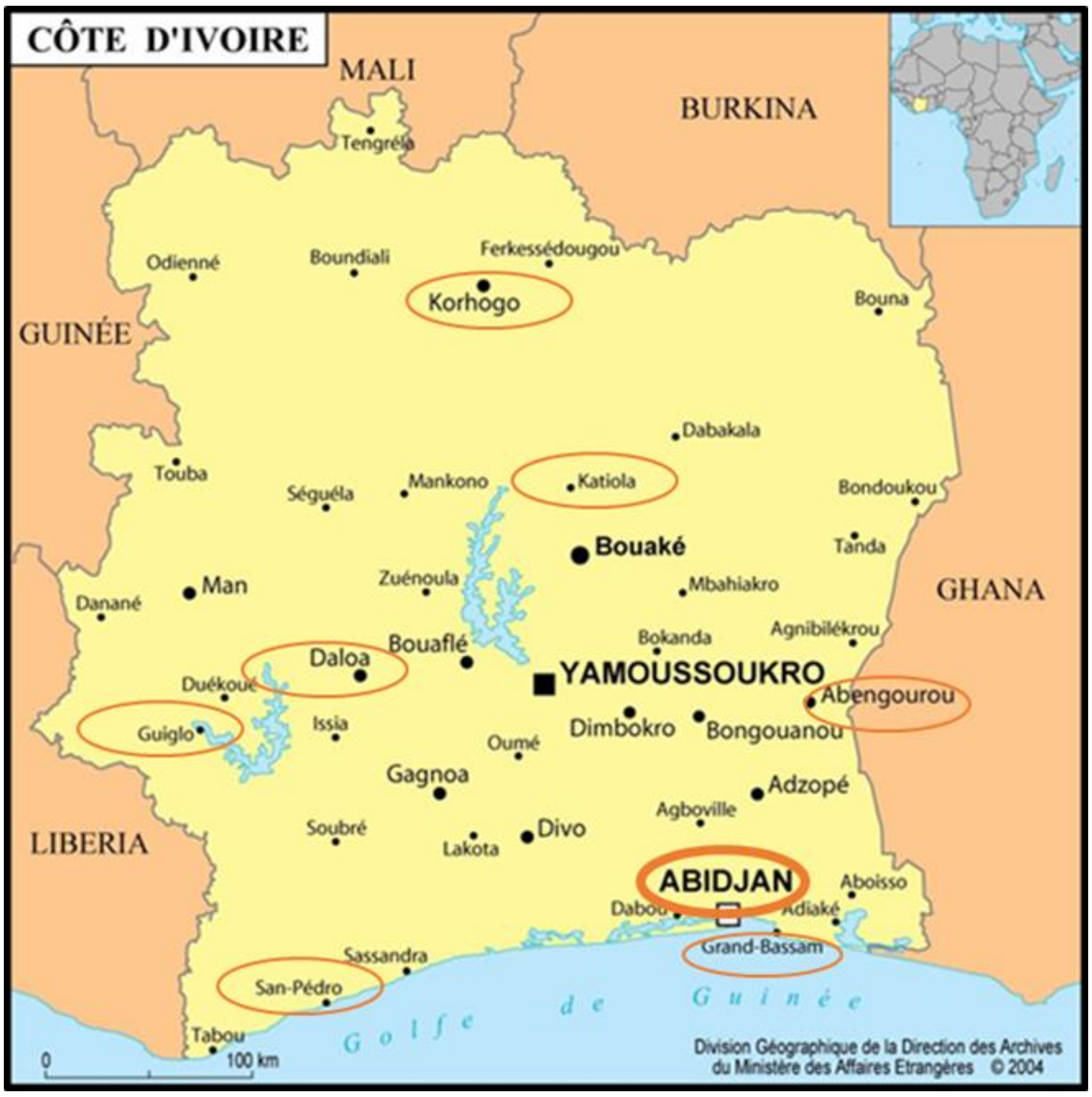

Supplementary file 1: Map of Côte d'Ivoire and List of participating cities

Supplementary file 2: List of key informants, Qualitative component

Category of informants	***Location***	***Age***	***Education level/Working status***	***Gender***	***Religion***
AGLHIV1	Abidjan	13	CE2 (2nd grade, elementary school)	Female	Muslim
AGLHIV2	Abidjan	19	University (1st year)	Female	Christian
AGLHIV3	Abidjan	18	6th year (middle school)	Female	Christian
AGLHIV4	Abidjan	18	3rd year (middle school)	Female	Muslim
AGLHIV5	Abidjan	17	High school	Female	Muslim
AGLHIV6	Abidjan	13	CM1 (*4th Grades* elementary school)	Female	Christian

AGLHIV7	Abidjan	19	University (first year)	Female	Christian
AGLHIV8	Abidjan	18	High school (last year)	Female	Christian
AGLHIV9	Abidjan	12	CM2 (*5th Grade* elementary school)	Female	Muslim
AGLHIV10	Abidjan	13	4th year (middle school)	Female	Christian
AGLHIV11	Abidjan	15	High school	Female	Christian
AGLHIV12	Abidjan	19	University (2nd year)	Female	NA
AGLHIV13	Abidjan	16	High school	Female	Christian
AGLHIV14	Abidjan	15	High school	Female	Christian
AGLHIV15	Abidjan	17	High school	Female	Muslim
AGLHIV16	Bouake	13	unschooled	Female	Muslim
AGLHIV17	Bouake	12	CM2 (*5th Grade* elementary school)	Female	Muslim
AGLHIV18	Abengourou	18	Unschooled	Female	Christian
AGLHIV19	Abengourou	16	Unschooled	Female	Christian
AGLHIV20	Daloa	12	CM2 (*5th Grade* elementary school)	Female	Muslim
AGLHIV21	Daloa	12	CM1 (*4th Grade* elementary school)	Female	Muslim
AGLHIV22	Daloa	15	Merchant	Female	Muslim
PLG 1	Abidjan	44	Housewife	Female	Muslim
PLG 2	Abidjan	42	Merchant	Female	Muslim
PLG 3	Abidjan	43	Housewife	Female	Muslim
PLG 4	Abidjan	47	Merchant (illiterate)	Female	Christian
PLG 5	Abidjan	21	High school	Male	Muslim
PLG 6	Abidjan	44	Policeman	Male	Christian
PLG 7	Abidjan	46	Esthetician	Female	Christian

PLG 8	Abidjan	55	Business owner	Male	Christian
PLG 9	Bouake	48	Merchant	Female	Muslim
PLG 10	Bouake	48	Merchant	Female	Muslim
PLG 11	Daloa	48	Merchant	Female	Muslim
PLG 12	Daloa	54	Merchant	Female	Muslim
PLG 13	Daloa	34	Merchant	Female	Muslim
**Healthcare professionals**					
HP 1	Abidjan	56	Pediatrician	Male	Christian
HP 2	Abidjan	53	Pediatrician	Female	Christian
HP 3	Abidjan	40	Professor, pediatrician	Female	Christian
HP 4	Abidjan	40	Pediatrician	Female	Christian
HP 5	Abidjan	39	Midwife	Female	Christian
HP 6	Bouake	39	Nurse assistant	Female	Christian
HP 7	Bouake	39	Community health worker	Female	Muslim
HP 8	Bouake	42	Physician	Male	Christian
HP 9	Abengourou	26	Nurse	Male	Muslim
HP 10	Abengourou	38	Nurse	Male	Christian
HP 11	Daloa	28	Nurse assistant	Female	Christian
**Policymakers**					
PM 1	Abidjan	47	Midwife, HIV program	Female	Christian
PM 2	Abidjan	48	Nurse, national program	Female	Christian
PM 3	Abidjan	63	Medical doctor, Cancer program	Female	Christian
PM 4	Abidjan	39	Public health specialist, implementing partner	Male	Christian
